# Hemodialysis-Related Amyloidosis in the Tongue

**DOI:** 10.1155/2022/9098201

**Published:** 2022-05-18

**Authors:** Satoshi Nakamura, Miki Yamada, Yosuke Iijima, Keisuke Sawada, Shunsuke Hino, Takahiro Kaneko, Norio Horie

**Affiliations:** ^1^Department of Oral and Maxillofacial Surgery, Saitama Medical Center, Saitama Medical University, Saitama, Japan; ^2^Department of Pathology, Saitama Medical Center, Saitama Medical University, Saitama, Japan

## Abstract

Dialysis-related amyloidosis (DRA) represents a group of relatively rare disorders characterized by the systemic extracellular deposition of insoluble fibrils of amyloid protein in long-term dialysis patients. We describe herein a case of relatively early DRA on the tongue of a long-term dialysis patient. A 67-year-old man with a 39-year history of dialysis was referred for diagnosis of a tongue mass. On examination, a collection of whitish-yellow papules was identified on the ventral surface of the tongue tip. The pathological diagnosis was DRA. Clinicians should be aware that long-term dialysis can cause oral amyloidosis of the tongue.

## 1. Introduction

In end-stage renal disease, as the final stage of chronic kidney disease, the kidneys no longer function and are thus unable to appropriately remove waste products or fluid from the blood. Chronic dialysis is the only option for patients with end-stage renal disease who are ineligible for renal transplantation [1, 2]. Amyloidosis represents a group of relatively rare disorders characterized by the extracellular deposition of insoluble fibrils of amyloid protein [3]. As a complication of long-term hemodialysis (HD), “dialysis-related amyloidosis” (DRA) represents the accumulation of amyloid fibrils from plasma concentrations of *β*2-microglobulin (*β*2m). DRA manifests first in the osteoarticular form and in some cases may appear peripherally on the skin and mucous membranes (tongue). Typical symptoms of DRA are carpal tunnel syndrome, bone cysts, scapula-humeral periarthritis, joint arthropathy, and destructive spondyloarthropathy [4]. DRA is rarely seen in the oral region but when present is found primarily on the tongue. Amyloid accumulation that becomes extensive may present as macroglossia [3].

Histopathologically, amyloid appears as amorphous eosinophilic extracellular deposits in hematoxylin and eosin staining. With Congo red stain, amyloid stains red under light microscopy and displays apple-green birefringence under polarized light. Other stains such as direct fast scarlet (DFS) can also confirm the presence of amyloid deposits [5].

Here, we report a case of relatively early-stage tongue amyloidosis in a long-term HD patient with systemic DRA.

## 2. Case Report

**Figure 1:**
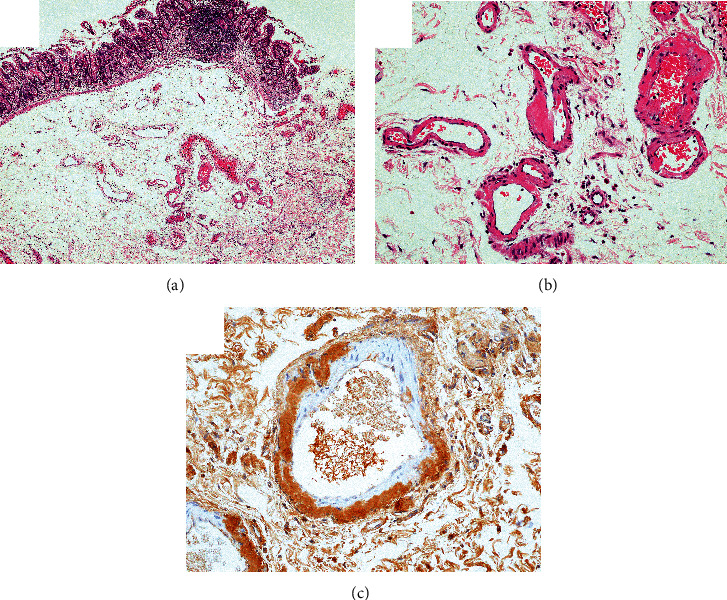
(a) A low-power view of the ileal biopsy specimen (hematoxylin and eosin stain; original magnification ×40). (b) Amorphous eosinophilic deposits are apparent on the vessel wall (hematoxylin-eosin stain, original magnification ×200). (c) Immunohistochemically, deposits show positive staining for *β*2-microglobulin (original magnification ×200).

**Figure 2:**
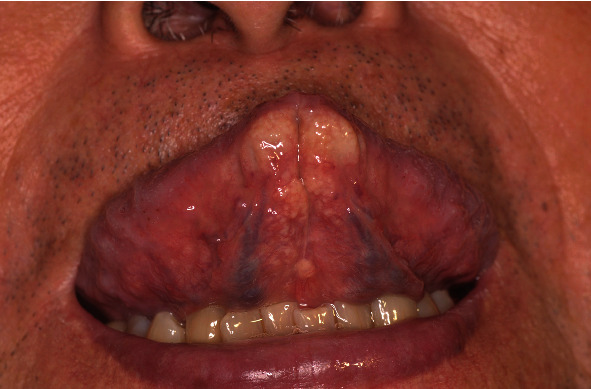
A collection of whitish-yellow papules is evident on the ventral surface of the tongue tip.

**Figure 3:**
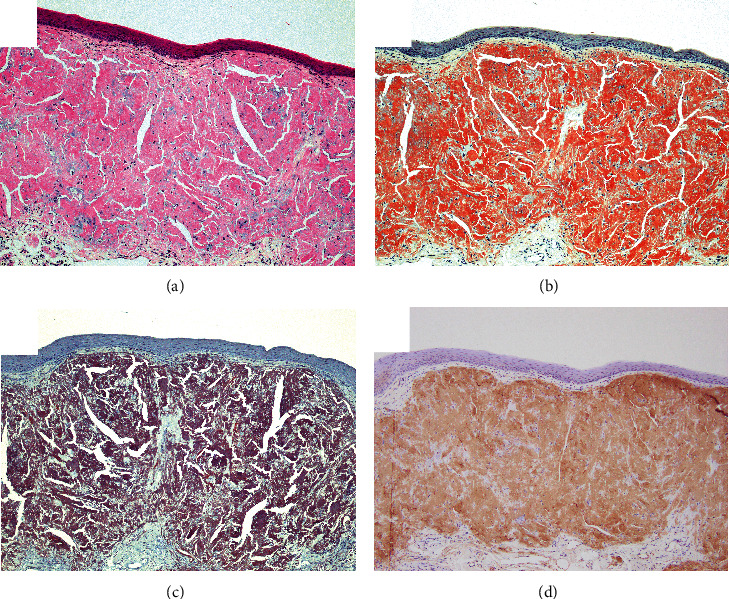
(a) Amorphous extracellular eosinophilic deposits are shown in the subepithelial region (hematoxylin-eosin stain, original magnification ×100). (b) Extracellular deposits show positive staining with direct fast scarlet (DFS) (original magnification ×100). (c) Extracellular deposits show positive staining with potassium permanganate- (KMnO_4_-) DFS stain (original magnification ×100). (d) Staining for *β*2-microglobulin is also positive (original magnification ×100).

The patient was a 67-year-old man who had been on HD for 39 years due to chronic renal failure. About 3 months earlier, he had noticed discomfort in the tongue and was referred by a dialysis doctor for examination. His medical history other than chronic renal failure (at 27 years old) included right nephrectomy for renal cell carcinoma (at 47 years old), left nephrectomy for renal cell carcinoma (at 50 years old), parathyroidectomy (at 51 years old), surgery for lumbar spinal stenosis (at 56 years old), surgery for destructive spondyloarthropathy (at 59 years old), right-side colon resection for ileus due to intestinal tuberculosis (at 63 years old), surgical decompression of carpal tunnel syndrome (at 66 years old), and ileal biopsy suggestive of amyloidosis (at 67 years old). Histopathological examination of the specimen from ileal biopsy showed amorphous eosinophilic deposits on the vessel wall (Figures 1(a) and 1(b)). Immumohistochemically, deposits were positive for *β*2m (Figure 1(c)). Current medications included alfacalcidol, ursodeoxycholic acid, sevelamer hydrochloride, and lanthanum carbonate hydrate, and the serum *β*2m level was 20.7 mg/L. In addition, the renal cell carcinomas and surgical decompression of carpal tunnel syndrome were not treated at the referring hospital and detailed data were not available. On oral examination, a collection of painless, relatively hard, rugged whitish-yellow papules was found on the ventral surface of the tongue tip (Figure 2). No movement disorder of the tongue was apparent. The patient showed no difficulty in eating or dysphagia. No abnormal findings were found in other sites of the oral cavity or regional lymph nodes. Biopsy was performed under local anesthesia. Histopathologically, the specimen showed amorphous eosinophilic extracellular deposits in the subepithelial region (Figure 3(a)). Extracellular deposits showed positive results from DFS staining, and also from potassium permanganate- (KMnO_4_-) DFS staining and *β*2m staining (Figures 3(b)–3(d)). The pathological diagnosis was amyloidosis, strongly suggestive of DRA. At the 6-month follow-up, no significant change in systemic DRA was seen, including tongue symptoms.

## 3. Discussion

Recent experimental findings have documented a direct cellular toxicity of *β*2m fibrils, but the mechanisms underlying *β*2m fibrillogenesis remain incompletely understood [6]. This syndrome can also be observed in end-stage renal diseases patients undergoing peritoneal dialysis and even in patients with chronic renal failure before the initiation of dialytic therapy, suggesting that HD is not a direct cause, but that accumulation of *β*2m or some *β*2m-associated molecules in the body is a common pathogenesis [7]. Although a high plasma concentration of *β*2m is associated with the development of DRA, other factors have been clearly incriminated, such as older age at dialysis onset and longer dialysis vintage or suspected effects such as proinflammatory effects of bioincompatible dialysis techniques [6].

DRA can be classified into two stages: initial and late. The initial stage recognizes osteoarticular DRA, and the late stage shows extra-articular DRA [8]. Oral amyloidosis belongs to the late stage, developing later than other DRAs. Cases of oral amyloidosis are thus uncommon. In oral DRA, the tongue is the most frequent site [8]. In addition to DRA, oral amyloid deposition can occur in primary amyloidosis, secondary amyloidosis, and localized amyloidosis. In general, with any amyloidosis, the tongue initially presents with whitish-yellow, relatively hard papules and nodules of varying amounts and sizes. Occasionally, functional disturbances of the tongue and dysgeusia are observed. In advanced cases, the tongue shows macroglossia, and eating disorders, respiratory disorders, and dysphagia may arise [3, 8]. Distinguishing DRA from other causes of amyloidosis may be difficult on macroscopic examination. In the present case, although tuberculosis could have been a cause of amyloidosis, the histological findings and dialysis history confirmed DRA by the identification of *β*2m. As the deposit was fairly localized on the ventral surface of the tongue tip, this might be an early symptom of DRA of the tongue.

Development of DRA is known to occur in almost 100% of cases within 15 years and is also related to the type of membrane and dialysate used. In this case, DRA was identified more than 25 years after starting dialysis. Over the last two decades, the prevalence and severity of DRA appear to have decreased significantly, although recent large-scale epidemiological studies show that DRA continues to be used [6]. Improved dialysis technology has definitely played a role in delaying the onset of disease [6]. In particular, the use of cellulosic low-flux membranes with different biocompatibilities was deeply involved in the retention of *β*2m [9]. This patient has recently been using a high-flux membrane. In addition, control of the clinical situation involves a regimen using a Lixelle adsorptive column, which uses porous cellulose beads (mean diameter, 460 *μ*m), the surface of which is covered with hydrophobic hexadecylalkyl chains in connection with a dialyzer [10, 11]. The use of ultrapure dialysate and the type of dialyzer membrane also help prevent the deposition of substances such as *β*2m [12].

Oral DRA previously has been estimated to occur in 20% of patients [8]. However, opportunities to observe oral DRA have been rare. This may also be due to decreases in DRA. In this case, oral DRA was found after more than 35 years of dialysis history.

Importantly, no current dialytic modality seems able to fully prevent DRA, and some patients have not received dialysis under proper dialysis conditions. Therefore, although the onset of DRA is delayed, DRA can still arise [6].

The differential diagnoses include various diseases that form a mass on the tongue and diseases that present as macroglossia. However, not so many diseases show a rugged mass with whitish-yellow coloration like dialysis amyloidosis. Lipomas and verruciform xanthoma are yellowish but are soft and do not feel rugged. In addition, lipomas may present with multiple nodules, and verruciform xanthoma may display a granular appearance [13]. Biopsy is evidently required to confirm the diagnosis, and the history of dialysis is extremely important for the diagnosis of DRA.

No curative treatments are yet available for DRA. In case of DRA of the tongue, palliative surgical reduction may be performed in cases with severe complications [3]. Preventing the development of DRA may require the implementation of improved dialysis technology, including biocompatible high-flux membranes and ultrapure dialysate [9].

In conclusion, DRA has not disappeared altogether. General dentists and oral surgeons should be aware that long-term dialysis can cause oral amyloidosis of the tongue.
